# Practical Hints to Those Who Dissect Human Bodies for Judicial Purposes

**Published:** 1855-02

**Authors:** 


*Practical Hints to those who Dissect Human Bodies for Judicial Purposes.*—I present to the consideration of my professional brethren, a few brief notes, of post-mortem examinations, made for the coroner of the city and county of Philadelphia. I shall attempt, after a service of some eighteen years in this department, to lay down a sort of chart for the young anatomist, to direct him in tracing out the cause of death, in the bodies of those whom he may be called to examine for judicial or other purposes. I would recommend at the outset of all these cases, that the fees for the service be determined upon beforehand. This should be done to avoid all future misunderstandings; and justice to the operators and the profession, the latter can never command the respect of the community until it is properly recommended. Above all things let the young medical men never forget to be *just* before God and man, in giving an opinion. Be careful how the opinion is formed, and never forget that mercy is one of the divine attributes, and under all circumstances, to give the accused benefit of every reasonable doubt as to the cause of death. Recollecting that, however much skill the medical men may possess, that there are cases of sudden death which he may be called upon to examine, where he will be satisfied that no *cause* can be demonstrated. Every part of the organization will appear in a perfectly healthy condition.

The first thing to be done in the examination is to mark (if buried) the character of the soil, the state of the weather, the hour of the day, and the date of the month and year that the examination takes place. These are to be carefully noted in a book for the purpose. If the body be not buried, its position at the time; the clothing, in detail; whether bloody or not, or torn, or otherwise. Clothes are to be taken off, observing any wounds or bruises, being cautious not to confound discolorations of the skin, arising of the blood gravitating from the most dependent parts, as the result from external violence. The condition of the pupils, whether dilated or contracted, regular or irregular; the mouth, anus and ears, (for through the latter, it is said that Hamlet's father met his death in the garden, while sleeping,) that no injuries have been received, or that they contain any foreign bodies.

Secondly, dissect carefully the head, chest, and the abdomen, finishing with the latter, but respect the dead by not resorting to any unnecessary mutilations of the parts, and thereby wounding the feelings of the living: never hesitate, however, when circumstances demand it, to destroy every vestige of the body, when necessary for the ends of justice. Let not your neglect to open any cavity of the body be a source of difficulty to medical men, who may not have been present at the autopsy, in forming their opinion. The evil of this neglect is plainly illustrated in the case of Sir John Dollen, who was tried and executed in London, for the murder of Sir Theodocious Broughton, by giving him laurel water, from the effects of which he was supposed to have died. There were five physicians for the Crown, and *one* for the defence—that one was the illustrious John Hunter. All the witnesses testified that Sir Theodocious had a large head and neck. No examination of the head had been made, I am clearly of the opinion that an innocent man suffered capital punishment from the over-zeal of the members of our profession. I again caution you, at all times, and under all circumstances, to be merciful and to be just. Let no vain feeling of display enter your breast when before the legal tribunals of your country. While giving your testimony in cases of homicide, a thoughtless word or act on your part may contribute to send an innocent man to a felon's cell or to the scaffold. State the facts of the case clearly, your own inference, and the reason on which the inference is founded, in a straightforward manly way. Do not be thrown off your guard by the artful questions of designing advocates. Be true to your conscience, and not be led from the statement of plain facts and honest opinions.—*Philadelphia Med. and Surg. Jour.*

